# Looking through the 'window of opportunity': is there a new paradigm of podiatry care on the horizon in *early *rheumatoid arthritis?

**DOI:** 10.1186/1757-1146-3-8

**Published:** 2010-05-17

**Authors:** James Woodburn, Kym Hennessy, Martijn PM Steultjens, Iain B McInnes, Deborah E Turner

**Affiliations:** 1 Musculoskeletal Rehabilitation Research Group, Institute of Applied Health Research, School of Health, Glasgow Caledonian University, Cowcaddens Road, Glasgow G4 0BA, UK; 2 Glasgow Biomedical Research Centre, University of Glasgow, 120 University Place Glasgow, G12 8TA, UK

## Abstract

Over the past decade there have been significant advances in the clinical understanding and care of rheumatoid arthritis (RA). Major paradigm changes include earlier disease detection and introduction of therapy, and 'tight control' of follow-up driven by regular measurement of disease activity parameters. The advent of tumour necrosis factor (TNF) inhibitors and other biologic therapies have further revolutionised care. Low disease state and remission with prevention of joint damage and irreversible disability are achievable therapeutic goals. Consequently new opportunities exist for all health professionals to contribute towards these advances. For podiatrists relevant issues range from greater awareness of current concepts including early referral guidelines through to the application of specialist skills to manage localised, residual disease activity and associated functional impairments. Here we describe a new paradigm of podiatry care in early RA. This is driven by current evidence that indicates that even in low disease activity states destruction of foot joints may be progressive and associated with accumulating disability. The paradigm parallels the medical model comprising early detection, targeted therapy, a new concept of tight control of foot arthritis, and disease monitoring.

'Podiatrists are experts on foot disorders: both patients and rheumatologists can profit from the involvement of a podiatrist'

- Korda and Balint, 2004 [1].

## Early RA

There is no established definition for *early *rheumatoid arthritis. Historic criteria for the classification of RA such as the American College of Rheumatology classification criteria are based on patients with long-standing disease. These criteria lack sensitivity in early disease and delaying treatment until patients fulfil such criteria is no longer acceptable. By symptom duration, the definition of early RA has progressively shorted from <5 years to <12-24 months, whilst *very early *disease indicates the period within the first 12-16 weeks of symptoms [2]. In practice early arthritis is often undifferentiated and may go on to remission, develop into established RA or other form of arthritis, or remain undifferentiated [3-5]. The imminent introduction of the new EULAR/ACR diagnostic criteria for RA will substantially improve matters in the medium term. Meantime, the clinical challenge in early disease is to recognise inflammatory arthritis, exclude diseases other than RA, estimate the risk of patients developing persistent, erosive, and irreversible disease, and to initiate therapy and thereafter monitor disease for optimal outcome [6].

### Advances in early RA

Understanding of rheumatoid arthritis has undergone a revolution in the past two decades in clinical and discovery domains [3]. Notably, concepts of the pathogenesis of RA have evolved considerably in this period, leading directly to introduction of biological therapeutics [6]. The development of an optimal strategic approach includes the early use of traditional disease-modifying anti-rheumatic drugs (DMARDs) and prompt advent of biologic based interventions in appropriate patients. In consequence, outcomes that can now be achieved are significantly advanced. A key message from recent research is the requirement for rapid recognition and early 'aggressive' intervention. Consider the following evidence, that;

- Ultrasound (US) and magnetic resonance imaging (MRI) studies demonstrate erosive changes from the early stages of RA [7-9].

- Functional loss occurs early and once present is often irreversible [5].

- Mortality rates for RA are increased [10].

- A biological 'window of opportunity' probably exists whereby intervention can alter the ultimate pathogenetic fate for the disease, leading to improved outcomes [11-14]. This is supported by evidence which indicates that early introduction of most treatment modalities is associated with improved clinical response rates. Early intervention with potent biological agents appears to offer profound improvements in clinical response rates and in the magnitude of benefit. A modest proportion of patients may achieve subsequent drug free periods of remission. A new strategy in early RA called 'tight control' aims for remission and tailors the treatment strategy to individual patients' disease activity [15-17]. Tight control is achieved by regular monitoring using composite, largely objective disease activity indices, the components of which capture both joint damage and functional impairment. Finally, good clinical practice indicates that it is difficult to justify delay in treating inflammatory disease once it is recognised.

### Foot involvement in early RA

Small joint arthritis is a hallmark feature of early RA and the feet are frequently involved at onset. Evidence to support this is taken from prospective and retrospective cohort studies which estimate the prevalence to be between 35-70% [18-20]. Prevalence is also high in all presenting inflammatory arthritis sub-types. In a *very early *arthritis cohort of 634 patients with symptoms ≤ 16 weeks duration, the ankle joint (18.9%) was the second most frequently involved joint after the knee (47.3%) in those with monoarthritis. In oligoarthritis (2-4 joints affected) the distribution of joint involvement was also high for the feet including ankle (43.5%), tarsus (7.9%), metatarsophalangeal (MTP) joints (18.1%) and toe joints (6.0%). In those patients with polyarthritis (≥ 5 joints affected), 50.3% had involvement of the MTP joints, 33.7% the ankle, 17.7% the tarsus and 9.7% the toe joints [2]. In a cohort of UK RA patients with <2 years duration, 90% of patients had experienced foot pain at some point of their illness [20].

Synovitis is detected clinically by joint swelling and effusion. Pain and tenderness indicates soft-tissue and structural joint damage, the consequence of inflammation, which is best detected and graded using plain x-ray or US. In the forefoot, van der Leeden *et al *(2008) found that 70% of patients with RA had pain and swelling of at least one MTP joint at diagnosis, decreasing to between 40-50% after two years with commencement of DMARD therapy [19]. However, both the prevalence and severity of forefoot joint damage progressively increased in this cohort over 8 years of follow up (prevalence 19% at baseline increasing to 60% and mean forefoot erosion score 1.3 at baseline increasing to 7.9).

Even patients in disease remission (based on the 28 joint count disease activity score - DAS28) may still have residual active disease in the feet. Discordance between DAS and DAS28 remission has been attributed to activity (tenderness and swelling) in the ankle and foot joints [21]. van der Leeden *et al *(2010) has shown that in 848 patients with recent onset RA, those reaching the DAS28 <2.6 remission criteria, 29% of cases had at least one painful MTP joint and 31% had at least one swollen MTP during an eight year follow up [22]. However, Kapral *et al *(2007) found higher patient global assessment of disease activity in patients with swollen and tender foot joints who were DAS28 inactive, concluding that assessment of the feet and ankles are important only in the clinical evaluation of patients with RA [23]. The reasons for localised disease persistence in the foot joints are unknown but mechanical factors have been postulated [24,25].

Retrospective radiographic studies suggest that involvement of the ankle and tarsus in early disease is rare with evidence of destructive changes observed in <1% of cases [26,27]. However, diagnostic MRI and US studies have been useful in detecting early synovitis in these joints as well as tendinopathies and bursitides, although none of these are epidemiological investigations [28-31]. Early involvement of the peritalar joints and tendinopathy of tibialis posterior in particular have been implicated in development of acquired pes planovalgus [24,32].

Foot associated functional impairment in early RA is poorly understood. van der Leeden *et al *(2008) estimated the prevalence of walking disability in an early arthritis cohort to be 57% [19]. Case-series data reveal the early stages of irreversible foot-related walking disability and, by detailed gait analysis, functional impairment at the ankle, tarsus and MTP joints [24].

What are the consequences of persistent or residual active foot disease which is not optimally managed? It is beyond the scope of this review to consider all the evidence but studies which span the paradigm shift in the clinical understanding of RA suggest high prevalence, high burden and an overall negative impact on quality of life. For example, a cross-sectional study of 1000 patients with established RA found that 80% of patients reported current foot problems and 71% reported difficulty in walking due to problems with their feet [18]. The prevalence of foot joint involvement did not differ between those in receipt of biological therapy (31%) and naïve patients. Highly prevalent features including pain history (90%), stiffness (77%), numbness (79%), and swelling (39%) have been reported in recent UK cohorts; and foot deformity (82-86%) and skin pressure lesions (79%) in Colombian and New Zealand cohorts [20,33,34]. Ultimately, foot-disease impacts negatively on health related quality of life [35].

### Non-pharmacological interventions for foot disease in early RA: evidence and guidelines

There is emerging evidence to suggest that multidisciplinary team care of patients with RA, including podiatry input, is effective in both inpatient and outpatient settings [36]. The specific contribution of podiatry may be unclear and difficult to separate as interventions such as insoles, splints and orthoses can be provided by other means, for example by an orthotist, physiotherapist or occupational therapist or bought over-the-counter by the patient. There is however a paucity of evidence for podiatry-led specialised foot care in early RA [36,37]. Only one randomised controlled trial of foot orthoses in relatively early disease has been published indicating that customised rigid foot orthoses, designed to control correctable rearfoot deformity and off-load painful joints, were more effective than standard orthoses prescribed under medical care for reducing foot pain and disability and restoring function [38,39]. This work has informed expert-led recommendations of several European groups [37,40]. For example, Gossec *et al *(2009) suggest that metatarsal pain and/or foot alignment abnormalities should be looked for regularly and that appropriate insoles should be prescribed if needed [37]. Forestier *et al *(2009) provide a disease-activity/staged non-pharmacological treatment strategy in which corrective orthoses are recommended after resolution of a flare, to restore functional range of motion and correct the level of physical activity [40]. In this protocol, preventative plantar insoles are recommended in stable early RA as part of a strategy to enable patients to accept their disease and prevent functional deterioration.

Despite this obvious lack of evidence, recommendations for foot care feature in many UK and European guidelines. These are summarised in Tables 1 and 2 for both early and established disease. Various recommendations are made for inclusion of podiatrists in the multidisciplinary care team, access to foot care, assessment and review, and various interventions including insoles, orthoses and footwear. However the level of supporting evidence is low, mainly at the 'good clinical practice' and 'expert opinion' agreement level. No reference to specialist podiatry assessment or extended scope practice could be found.

**Table 1:** Guidelines and recommendations for foot related non-pharmacological interventions in early rheumatoid arthritis.

	**Scottish Intercollegiate Guidelines Network Management of early rheumatoid arthritis **[69]	**Clinical practice guidelines for the use of non-pharmacological treatments in early rheumatoid arthritis **[37]	**British Society for Rheumatology and British Health Professionals in Rheumatology Guideline for the management of rheumatoid arthritis (the first 2 years) **[70]	**European League Against Rheumatism recommendations for the management of early arthritis **[71]	**Multidisciplinary guidelines for the management of early rheumatoid arthritis **[72]
**Multidisciplinary team care**	Podiatry is part of the multidisciplinary team		Podiatry is part of the multidisciplinary team Full-time dedicated podiatrist specialising in rheumatology is essential		Podiatry is part of the multidisciplinary team

**Access to foot health care**	'Good practice' to offer all patients with early RA a podiatry referral		Access to podiatry should be available according to patient need Podiatry services should provide specific and dedicated service for diagnosis, assessment and management of foot problems associated with RA Timely intervention for acute problems is important		Foot care can relieve pain, maintain function and improve quality of life

**Foot Health Assessment/Review**		Metatarsal pain and/or foot alignment abnormalities should be looked for regularly	Annual foot review/assessment is recommended for patients at risk of developing serious complications in order to detect problems early Appropriate lower limb assessment for vascular and neurological status is needed Assessment of lower limb mechanics and foot pressures should occur		Annual foot review is recommended for patients at risk of developing complications

**Orthoses/Insoles/Splints**	Some evidence for the efficacy of foot orthoses for comfort, and stride speed and length	Appropriate insoles should be prescribed if needed	Orthoses are an important and effective intervention in RA	Use of orthoses has shown short term relief of pain only, rather than an effect on disease activity.	Joint protection included-orthoses not specifically mentioned

**Therapeutic footwear**	Appropriate footwear for comfort, mobility, and stability is well recognised in clinical practice but little available evidence		There should be a provision of specialist footwear if needed		

**Table 2:** Guidelines and recommendations for foot related non-pharmacological interventions in established rheumatoid arthritis.

	**American College of Rheumatology Subcommittee on rheumatoid arthritis guidelines for the management of rheumatoid arthritis **[73]	**Arthritis and Musculoskeletal Alliance Standards of care for people with inflammatory arthritis **[74]	**Podiatry Rheumatic Care Association Standards of care for people with musculoskeletal foot health problems **[75]	**National Institute for Health and Clinical Excellence Rheumatoid arthritis National clinical guideline for management and treatment in adults **[76]	**British Society for Rheumatology and British Health Professionals in Rheumatology Guideline for the management of rheumatoid arthritis (after the first 2 years) **[77]	**Clinical Practice Guidelines for non-drug treatment (excluding surgery) in rheumatoid arthritis **[40]
**Multidisciplinary team care**		People with inflammatory arthritis should have ongoing access to local multidisciplinary team Podiatrists are part of the multidisciplinary team.	Early referral for surgical opinion if required			

**Access to foot health care**		All people with a sudden 'flare-up in their condition should have direct access to specialist advice and the option for early review with the appropriate multidisciplinary team member	Timely access to foot health care - diagnosis, assessment and management Adequate information/education should be given for self-management and signs/symptoms of deterioration in foot health and need to access specialist help promptly	All patients with RA and foot problems should have access to a podiatrist		Every patient with RA should be informed of the rules of foot hygiene and of potential benefit of referral to a podiatrist A podiatrist should be consulted to treat nail anomalies and hyperkeratoses on the feet of patients with RA

**Foot health assessment/review**			Foot health care providers must understand the consequences of systemic disease on the feet and be able to identify warning signs that require timely referral to specialist medical care Musculoskeletal foot health assessment should include: General health; Foot health; Systemic factors; Lifestyle/Social factors; Pain management; Need for other assessments as required Foot health assessment should occur within 3 months of diagnosis - doesn't have to be done by foot health specialist Annual review of foot health needs are desirable - doesn't have to be done by foot health specialist Where there is substantial change (better/worse) in disease activity, foot health should be reviewed	All patients with RA and foot problems should have access to a podiatrist for assessment and periodic review of their foot health needs		Feet, footwear and orthoses should be regularly examined

**Orthoses/Insoles/Splints**	Non-pharmacological treatment recommendations include joint protection but do not specifically mention orthoses			Functional insoles and therapeutic footwear should be available to all people with RA if indicated	Limited evidence for the use of foot orthoses - no consensus regarding choice of orthoses but reduction of pain and improved function of the foot are reported	Customised orthotic insoles are recommended in the case of weight-bearing pain or static foot problems Customised toe splints may be preventive, corrective or palliative to enable the wearing of shoes Orthoses should be regularly examined

**Therapeutic footwear**					Semi-rigid orthotic supportive shoes can be effective for metatarsalgia - reduction in pain, disability, and improvement in activity as measured by the Foot Function Index have been reported	Patients should be advised about footwear Footwear should be regularly examined Extra-width off-the-shelf or therapeutic shoes thermoformed on the patient's foot are recommended when the feet are deformed and painful, or if it is difficult to put on shoes - such shoes reduce pain on walking and improve functional capacity Off-the-shelf therapeutic thermoformed shoes for prolonged use are indicated when other types of footwear have failed Palliative customized therapeutic shoes may be prescribed when the feet are seriously affected

## A new paradigm for podiatry in early RA

What new opportunities do recent paradigm shifts in the management of early RA offer podiatrists? Evidence presented earlier indicates that active foot disease persists in many patients despite recent treatment advances. Moreover, access to biological therapy is variable, there are practical challenges to undertaking DAS28 monitoring in routine practice, and the required changes to service provision to accommodate new care pathways are barriers in translating evidence to practice [17,41,42]. Consequently, in clinical practice remission rates are around 20% depending on which criteria are used [43,44]. This evidence, combined with current guidelines and good clinical practice, indicates the need for ongoing multidisciplinary team care, including podiatry, in early RA.

Local development of this paradigm is based on experience from an academic-clinical partnership initiative in Glasgow, UK. Support for specialist podiatry training and professional development, clinical practice, and research and audit are jointly provided by academic rheumatology/podiatry units at The University of Glasgow and Glasgow Caledonian University in conjunction with National Health Service clinicians. This model is expanding in Scotland with knowledge transfer facilitated through the Podiatry Practice Development Group for Rheumatology, a National Health Services Quality Improvement Scotland Health Board network initiative for allied health professions. Key aspects of the paradigm include:

### Early detection - widespread dissemination and uptake of referral guidelines

The necessity to obtain specialist referral to guarantee early diagnosis and rapid treatment is evidenced by facts that structural damage occurs early in RA, that joint destruction increases the risk of irreversible disability, and that early introduction of most treatment modalities is associated with improved clinical response. The importance of clinical examination cannot be overlooked. Simple tests such as the MTP squeeze test are highly predictive of persistent erosive arthritis (outcome) and HAQ disability [33,45]. Recognising this, Emery and colleagues (2002) developed an early referral recommendation tool for primary care doctors (Appendix 1) [46]. Given the high prevalence of MTP joint involvement at onset, podiatrists should be aware of these guidelines when encountering patients with forefoot pain. Such patients can reach podiatrists through a number of referral routes with an initial diagnosis of mechanically-related metatarsalgia. The algorithm is easy to understand and apply and should be widely disseminated among podiatrists.

*Therefore, under this new paradigm we propose to increase the knowledge and understanding of early RA, including the mandate for early recognition and treatment. The early referral algorithm proposed by Emery et al (2002) should be brought to the attention of all podiatrists utilising national networks for dissemination and training *[46].

### Targeted therapy - aggressive management of residual foot disease

Recommendations for podiatry/foot care in early RA places an emphasis on access, annual review for those at risk of developing foot complications and timely interventions (Table 1). Currently, definition of need, risk, and timeliness are poorly understood. A pragmatic approach may be to identify three groups of patients. Firstly those with low disease activity (by DAS28) who have residual disease activity in the foot with associated impairment and disability. Case identification can be facilitated by raising awareness among the rheumatology multidisciplinary care team, including training sessions on foot problems, examination and management. Red flag conditions should be prioritised e.g., tibialis posterior tendinopathy with early flat-footedness, persistent synovitis in any of the tarsus joints and persistent, non-responsive and symptomatic forefoot disease despite low disease state/remission. Further work is required before evidence based recommendations can be made for routine screening of all early RA patients. The second group are those with medium to high disease states where personalised non-pharmacological interventions are undertaken based on the presenting impairments to act in conjunction with the systemic management. The third group are those patients who fail to respond to biological therapy or are ineligible and require close monitoring and care of active foot joints.

In our opinion, targeted foot care should be delivered by specialist podiatrists working in a multidisciplinary clinic in both primary and secondary care. Extended scope practice should include specialist training in diagnostic ultrasonography (using recognised training pathways, for example the PGcert in Medical Ultrasound); corticosteroid injection therapies; non-pharmacological interventions; gait analysis and rehabilitation. In the UK, multidisciplinary foot clinics in rheumatology are not new and they generally comprise of the podiatrist, extended-scope physiotherapist and orthotist [47,48]. The rheumatologist, nurse specialist and orthopaedic surgeon may be in attendance or a rapid referral pathway developed. Evidence for such an approach is lacking, but the area has been identified as a research priority.

A new paradigm for podiatrists focuses on *combination therapy *targeted at inflammatory lesions and associated mechanically-based impairments. This should include ultrasound-guided aspirations, intra-articular and soft-tissue corticosteroid injection therapy with cast immobilisation for residual lesions, and customised orthotics, exercise and gait training for associated impairments. Patients with evidence of joint instability and passively correctable deformities should be targeted with highly personalised orthotics, exploiting newer computer-aided design and manufacture capabilities where available. Orthotics treatment can be combined with exercises, gait training, and therapeutic footwear, as well as joint protection and disease management advice and support. Minor surgical procedures including nail surgery and cryosurgery are within the scope of practice for UK podiatrists. Bone, joint and soft-tissue surgery is restricted to those with advanced training and beyond the scope of this review. Important training issues related to current guidelines for RA patients in receipt of biological and other immune system suppressing medication should be provided during training [49]. UK podiatrists also have limited prescribing rights and within the multidisciplinary clinic for early RA, access is generally limited to analgesic, corticosteroid and antibiotic medicines.

Podiatrists should also be trained to recognise and appropriately refer disease flare and other associated complications. This includes, for example, skin and nail infections of the feet in patients receiving biological therapy as previously reported [50,51]. Podiatrists also possess core skills to assess and monitor peripheral vascular and neurological diseases. Routine techniques such as ankle-brachial pressure indices can be applied to screen for potential risk factors for cardiovascular disease [52].

*Under this paradigm we propose that specialist podiatry roles are created, supported by high-level training and mentorship, and that podiatrists actively engage in Early Arthritis Clinics as part of the multidisciplinary team. Accordingly patients should be targeted and treated aggressively using injection therapy and personalised rehabilitation interventions, with appropriate referral where indicated*.

### Tight control of foot arthritis and disease monitoring

The concept of tight control can be applied for peripheral joints as a central paradigm for podiatrists to aim for the lowest foot disease state or remission. Care can then be escalated or tapered based on monitoring foot disease and related impairment and disability using a number of clinical metrics. These are summarised in Table 3 as candidate outcomes for core and extended datasets. These span foot-specific disease activity, joint destruction, and impairment and disability, with a balance of objective and patient-orientated outcomes. Swollen and tender joint counts are based on the Ritchie Articular Index which originally incorporated the tibio-talar, subtalar, midtarsal, MTP and interphalangeal joints [53]. The Structural Index is a semi-objective scale to measure foot deformity and function. It works adequately in practice but requires validation [54]. The Foot Function Index (FFI) and Foot Impact Scale (FIS) for RA are well-validated RA specific outcome tool for foot related impairment and disability [55,56]. The psychometric properties of both instruments currently make them the most appropriate outcome instruments to determine treatment escalation or tapering [57]. Routine monitoring by DAS28 has led to larger numbers of patients reaching low disease state through increased changes in DMARD treatment [58]. On that basis use of the FFI and FIS must be promoted among podiatrists to drive treatment change and provide objective treatment targets.

**Table 3:** Candidate outcome for core and extended clinical foot datasets.

Outcome	Domain
**CORE**	
1. Swollen foot joint count	Active disease
2. Tender foot joint count	Joint destruction/soft-tissue damage
3. Foot Impact Scale-RA	Foot impairment and disability
4. Structural Index	Foot deformity
5. Radiographic erosions	Joint destruction
**EXTENDED**	
6. Ultrasound core set	Active disease/joint destruction Soft-tissue disease
7. Gait analysis - spatiotemporal, plantar pressure, joint motion and forces	Functional

Radiographic erosions, scored using Sharp-van der Heijde method should be reviewed during routine follow-up. Within extended scope practice, B-Mode and power Doppler ultrasound (US) is being increasingly used by specialist podiatrists. The advantages, especially for inflammatory foot disease are well established and the clinical utility for podiatrists is extremely high. US permits better identification of synovitis and erosions in foot joints over conventional radiographs in early disease [59-61]. US is superior to clinical examination for locating and quantifying synovitis, erosions and tendinopathies especially in complicated anatomical areas such as the peri-talar region [31,62-65]. Moreover, US has been shown to beneficially influence the planning of local corticosteroid injection therapy in the foot; to provide more accurate needle tip placement and subsequent injection as well as aspiration and infiltration of tendon sheaths, joint spaces and bursae. It lessens procedural pain and it leads to improved short-term efficacy [64,66,67]. Evidence is emerging of good competency standards among UK podiatrists undertaking US scanning techniques [68]. In an extended data set, and where access is available, three-dimensional gait analysis provides the most objective way of capturing functional changes in the foot. It has been successfully employed in early RA to detect subtle but clinically important functional changes [24].

Past experience from multidisciplinary foot clinics in rheumatology indicate that patients should be regularly followed up until problems are resolved [47]. In early RA patients this should constitute low foot disease state or remission, with concomitant improvements in impairment, related disability and quality of life. Early detection and aggressive treatment within a therapeutic window of opportunity when disability is potentially reversible is critical.

*Under this paradigm, we propose that podiatrists tightly control foot arthritis using personalised treatment plans which are agreed within the multidisciplinary team. Disease management should be escalated or tapered according to defined criteria combining objective image-based techniques and patient orientated outcomes*.

## Conclusions

Proposals contained within this commentary are predicated upon major developments in the clinical understanding of RA and paradigm shifts concerning early detection and treatment, tight control of disease and monitoring, and the introduction of biological therapies. However, despite these advances evidence indicates that active disease in the foot is an ongoing problem in clinical practice. A new paradigm of podiatry care can adopt these advancements in early disease, exploiting the extended scope practice capabilities and training opportunities available. To evidence the paradigm, UK podiatrists are forming multi-centre research networks to facilitate cohort and interventions studies. Studies are in progress to understand disease mechanisms, to assess the burden and impact of foot disease in early RA, to develop a minimum foot core-set, to define foot disease remission, and to pilot interventions, outcomes and health economic impact. These studies are building towards a definitive trial of the clinical and cost-effectiveness of foot care in early RA. If proven, the paradigm may be generalisable to other forms of inflammatory, post-traumatic and degenerative disorders in the musculoskeletal field as well as a model for the management of neurologic induced dysfunction, e.g., neuropathic ulceration and Charcot's disease.

## Competing interests

The authors declare that they have no competing interests.

## Authors' contributions

The authors conceived of the study and undertook the written review equally. KH led the electronic literature searches. All authors read and approved the final manuscript.

## Appendixes

**Appendix 1**. Early referral guidelines for newly diagnosed rheumatoid arthritis (after Emery *et al *2002) [46].

Rapid referral to a rheumatologist advised in the event of *clinical suspicion of RA*, which may be supported by the presence of any of the following:

≥ 3 swollen joints

MTP/MCP involvement- Squeeze test positive

Morning stiffness of ≥ 30 minutes
